# Supplementary material for: Metabolic plasticity can amplify ecosystem responses to global warming
Source: Nat Commun. 2022 Apr 20;13:2161. doi: 10.1038/s41467-022-29808-1 (PMC9021271; doi:10.1038/s41467-022-29808-1)
Supplement: Supplementary file 1 — Supplementary Information [file 41467_2022_29808_MOESM1_ESM.pdf]

Supplementary Information: Metabolic plasticity can amplify ecosystem responses to global warming

Rebecca L. Kordas[1], Samraat Pawar[1], Dimitrios-Georgios Kontopoulos[2,3], Guy Woodward[1], and Eoin J. O'Gorman[4,*]

[1] *Department of Life Sciences, Imperial College London, Silwood Park Campus, Buckhurst Road, Ascot, Berkshire, SL5 7PY, UK.*

[2] *LOEWE Centre for Translational Biodiversity Genomics, Senckenberganlage 25, 60325, Frankfurt, Germany*

[3] *Senckenberg Research Institute, Senckenberganlage 25, 60325, Frankfurt, Germany*

[4] *School of Life Sciences, University of Essex, Wivenhoe Park, Colchester, CO4 3SQ, UK.*

Running head: Metabolic plasticity and warming

Type of paper: Letter

*** Corresponding author:** Eoin O'Gorman

E-mail: e.ogorman@essex.ac.uk

Supplementary Discussion

Quantitative differences between energy fluxes and empirical ecosystem respiration

There is a strong positive relationship between our model-predicted sum of energy fluxes through all trophic links and empirical measurements of ecosystem respiration (Supplementary Figure 6; $r^2 > 0.70$). However, there are two quantitative differences between the observed and predicted values: the observed ecosystem respiration is approximately two orders of magnitude higher than the predicted total flux, and the slope of the relationship is 0.64, rather than 1 (i.e. the magnitude of the difference decreases at higher predicted energy flux values). These differences are expected because total ecosystem respiration does not equate to total energy flux through trophic links. Specifically: (i) the observed ecosystem respiration reflects the approximately 90% of energy lost due to inefficient transfer through food web links[1]; (ii) The observed ecosystem respiration also includes microbial and algal respiration, which are not included in our model (only losses by consumer species need to be balanced – see *Methods*[2]); (iii) The deviation from a 1:1 prediction (the sublinear slope of the observed and predicted relationship) likely stems from the invertebrate consumers accounting for a greater proportional biomass in the warmest streams[3] (i.e. invertebrate biomass increases, and diatom biomass decreases with increasing stream temperature). As a result, a greater proportion of ecosystem respiration is accounted for by our modelled energy flux (which is most strongly influenced by invertebrate metabolic rates) at higher temperatures, and the slope of the line deviates from 1 (i.e. gets closer to the empirical estimate) in the warmer streams.

Supplementary Note

R code for statistical model fitting

We followed the methods detailed by Zuur *et al*.[4] for determining the optimal random and fixed effects structures describing the effects of body mass, acute temperature exposure, and chronic temperature exposure on metabolic rate. Specifically, we started with the full model detailed in Equation 3 of the main text and determined the optimal random effects structure using the following R code:

```
lmec = lmeControl(opt = "optim", msMaxIter = 200, msMaxEval = 500))
r1 = gls(lnI ~ lnM + TA + TC + lnM:TA + lnM:TC + TA:TC + lnM:TA:TC,
                method="REML", control=lmec)
r2 = lme(lnI ~ lnM + TA + TC + lnM:TA + lnM:TC + TA:TC + lnM:TA:TC,
                random = ~1 | species,
                method="REML", control=lmec)
r3 = lme(lnI ~ lnM + TA + TC + lnM:TA + lnM:TC + TA:TC + lnM:TA:TC,
                random = ~1 + lnM | species,
                method="REML", control=lmec)
r4 = lme(lnI ~ lnM + TA + TC + lnM:TA + lnM:TC + TA:TC + lnM:TA:TC,
                random = ~1 + TA | species,
                method="REML", control=lmec)
r5 = lme(lnI ~ lnM + TA + TC + lnM:TA + lnM:TC + TA:TC + lnM:TA:TC,
                random = ~1 + TC | species,
                method="REML", control=lmec)
r6 = lme(lnI ~ lnM + TA + TC + lnM:TA + lnM:TC + TA:TC + lnM:TA:TC,
                random = ~1 + lnM + TA | species,
                method="REML", control=lmec)
r7 = lme(lnI ~ lnM + TA + TC + lnM:TA + lnM:TC + TA:TC + lnM:TA:TC,
                random = ~1 + lnM + TC | species,
                method="REML", control=lmec)
r8 = lme(lnI ~ lnM + TA + TC + lnM:TA + lnM:TC + TA:TC + lnM:TA:TC,
```

```
                random = ~1 + TA + TC | species,
                method="REML", control=lmec)
r9 = lme(lnI ~ lnM + TA + TC + lnM:TA + lnM:TC + TA:TC + lnM:TA:TC,
                random = ~1 + lnM + TA + TC | species,
                method="REML", control=lmec)
AIC(r1,r2,r3,r4,r5,r6,r7,r8,r9)
```

Here, ln$I$, ln$M$, T_A, and T_C were continuous variables defined in Equations 1–3 in the main text, while '*species*' was a categorical variable corresponding to the species name for each data point in the analysis. The values we used for '*lmeControl*' were simply to deal with convergence issues and were implemented consistently throughout our analyses. The model `r9` was determined to contain the optimal random effects structure using Akaike Information Criterion (ΔAIC > 31.2; see Supplementary Table 7). The subsequent R code for determining the optimal fixed effects structure was as follows:

```
m1 = lme(lnI ~ lnM + TA + TC + lnM:TA + lnM:TC + TA:TC + lnM:TA:TC,
                random = ~1 + lnM + TA + TC | species,
                method="ML", control=lmec)
m2 = lme(lnI ~ lnM + TA + TC + lnM:TA + lnM:TC + TA:TC,
                random = ~1 + lnM + TA + TC | species,
                method="ML", control=lmec)
m3 = lme(lnI ~ lnM + TA + TC + lnM:TA + lnM:TC,
                random = ~1 + lnM + TA + TC | species,
                method="ML", control=lmec)
m4 = lme(lnI ~ lnM + TA + TC + lnM:TA + TA:TC,
                random = ~1 + lnM + TA + TC | species,
                method="ML", control=lmec)
m5 = lme(lnI ~ lnM + TA + TC + lnM:TC + TA:TC,
                random = ~1 + lnM + TA + TC | species,
                method="ML", control=lmec)
m6 = lme(lnI ~ lnM + TA + TC + lnM:TA,
```

```
                random = ~1 + lnM + TA + TC | species,
                method="ML", control=lmec)
m7 = lme(lnI ~ lnM + TA + lnM:TA,
                random = ~1 + lnM + TA + TC | species,
                method="ML", control=lmec)
m8 = lme(lnI ~ lnM + TA + TC + lnM:TC,
                random = ~1 + lnM + TA + TC | species,
                method="ML", control=lmec)
m9 = lme(lnI ~ lnM + TC + lnM:TC,
                random = ~1 + lnM + TA + TC | species,
                method="ML", control=lmec)
m10 = lme(lnI ~ lnM + TA + TC + TA:TC,
                 random = ~1 + lnM + TA + TC | species,
                 method="ML", control=lmec)
m11 = lme(lnI ~ TA + TC + TA:TC,
                 random = ~1 + lnM + TA + TC | species,
                 method="ML", control=lmec)
m12 = lme(lnI ~ lnM + TA + TC,
                 random = ~1 + lnM + TA + TC | species,
                 method="ML", control=lmec)
m13 = lme(lnI ~ lnM + TA,
                 random = ~1 + lnM + TA + TC | species,
                 method="ML", control=lmec)
m14 = lme(lnI ~ lnM + TC,
                 random = ~1 + lnM + TA + TC | species,
                 method="ML", control=lmec)
m15 = lme(lnI ~ TA + TC,
                 random = ~1 + lnM + TA + TC | species,
                 method="ML", control=lmec)
m16 = lme(lnI ~ lnM,
                 random = ~1 + lnM + TA + TC | species,
```

```
                 method="ML", control=lmec)
m17 = lme(lnI ~ TA,
                 random = ~1 + lnM + TA + TC | species,
                 method="ML", control=lmec)
m18 = lme(lnI ~ TC,
                 random = ~1 + lnM + TA + TC | species,
                 method="ML", control=lmec)
m19 = lme(lnI ~ 1,
                 random = ~1 + lnM + TA + TC | species,
                 method="ML", control=lmec)
AIC(m1,m2,m3,m4,m5,m6,m7,m8,m9,m10,m11,m12,m13,m14,m15,m16,m17,m18,m19)
```

The model `m5` was determined to contain the optimal fixed effects structure (see Supplementary Table 8). Note that model `m2` performed similarly (ΔAIC = 0.2), but contained a non-significant interaction between ln$M$ and T_A (t_{1286} = -1.43, p = 0.1534). Thus, we also arrived at `m5` by dropping each non-significant higher order term until all terms in the model were significant[4]. The R code for exploring the best-fitting model was thus:

```
summary(mod = lme(lnI ~ lnM + TA + TC + lnM:TC + TA:TC,
                        random = ~1 + lnM + TA + TC | species,
                        method="REML", control=lmec))
```

R code for plotting the partial residuals

To visualise the effect of chronic temperature exposure on the size-dependence of metabolic rate (Fig. 1a), we first had to temperature-correct the metabolic rate data according to the acute temperature exposure parameters in our best fitting model. We then refitted the model to the temperature-corrected data, removing any terms in the model containing acute temperature exposure. Finally, we extracted the partial residuals for plotting as follows:

```
tcor_I = I / exp(TA*(summary(mod)$tTable["TA","Value"] +
        summary(mod)$tTable["TA:TC","Value"]*TC))
```

```
tcor_mod = lme(ln(tcor_I) ~ lnM + TC + lnM:TC,
                             random = ~1 + lnM + TC | species,
                             method="REML", control=lmec))
tcor_resids <- resid(tcor_mod)
tcor_partials = tcor_resids +
                 summary(tcor_mod)$tTable["(Intercept)","Value"] +
                 summary(tcor_mod)$tTable["lnM","Value"]*lnM +
                 summary(tcor_mod)$tTable["TC","Value"]*TC +
                 summary(tcor_mod)$tTable["lnM:TC","Value"]*lnM*TC
```

Similarly, to visualise the effect of chronic temperature exposure on the acute temperature-dependence of metabolic rate (Fig. 1c), we first had to mass-correct the metabolic rate data according to the body mass parameters in our best fitting model. We then refitted the model to the mass-corrected data, removing any terms in the model containing body mass. Finally, we extracted the partial residuals for plotting:

```
mcor_I = I / M^(summary(mod)$tTable["lnM","Value"] +
          summary(mod)$tTable["lnM:TC","Value"]*TC)
mcor_mod = lme(ln(mcor_I) ~ TA + TC + TA:TC,
                             random = ~1 + TA + TC | species,
                             method="REML", control=lmec))
mcor_resids <- resid(mcor_mod)
mcor_partials = mcor_resids +
                 summary(mcor_mod)$tTable["(Intercept)","Value"] +
                 summary(mcor_mod)$tTable["TA","Value"]*TA +
                 summary(mcor_mod)$tTable["TC","Value"]*TC +
                 summary(mcor_mod)$tTable["TA:TC",″Value"]*TA*TC
```

To visualise the effect of chronic temperature exposure on the allometric exponent, *b* (Fig. 1b), we performed a generalised least squares regression on the residuals of our temperature-corrected model (i.e. ‘`tcor_resids`’) to estimate how a data point deviates in dependence of

stream identity ('*streamID*'), nested within the deviance in the size-dependence (ln*M*). Similarly, to visualise the effect of chronic temperature exposure on the activation energy, *E* (Fig. 1d), we performed a generalised least squares regression on the residuals of our mass-corrected model (i.e. '`mcor_resids`') to estimate how a data point deviates in dependence of stream identity, nested within the deviance in the acute temperature-dependence (T_A). In both cases, we set the intercept to zero by including '-1' in the model description, following standard protocols for analysis of residuals[3,5]. Note that this was not a formal statistical analysis and was used solely for visualisation purposes. The R code for this procedure was as follows:

```
b_mod = gls(tcor_resids ~ streamID/lnM - 1, method="ML", data=final)
b_partials = summary(tcor_mod)$tTable["lnM","Value"] +
             summary(tcor_mod)$tTable["lnM:TC","Value"]*TC +
             summary(b_mod)$tTable[10:18]
E_mod = gls(mcor_resids ~ streamID/TA - 1, method="ML", data=final)
E_partials = summary(mcor_mod)$tTable["TA","Value"] +
             summary(mcor_mod)$tTable["TA:TC","Value"]*TC +
             summary(E_mod)$tTable[10:18]
```

Supplementary Figure 1. Map of the Hengill geothermal valley. Mean stream temperatures from May 2015 to July 2017 are shown, along with stream codes corresponding to previous publications from the study system[3,6,7]. Streams from which metabolic rates (and energy fluxes) were measured are coloured by their mean temperatures, with additional streams used in the exploration of energy flux coloured in light grey. A detailed temperature profile for each stream is shown in Supplementary Figure 2.

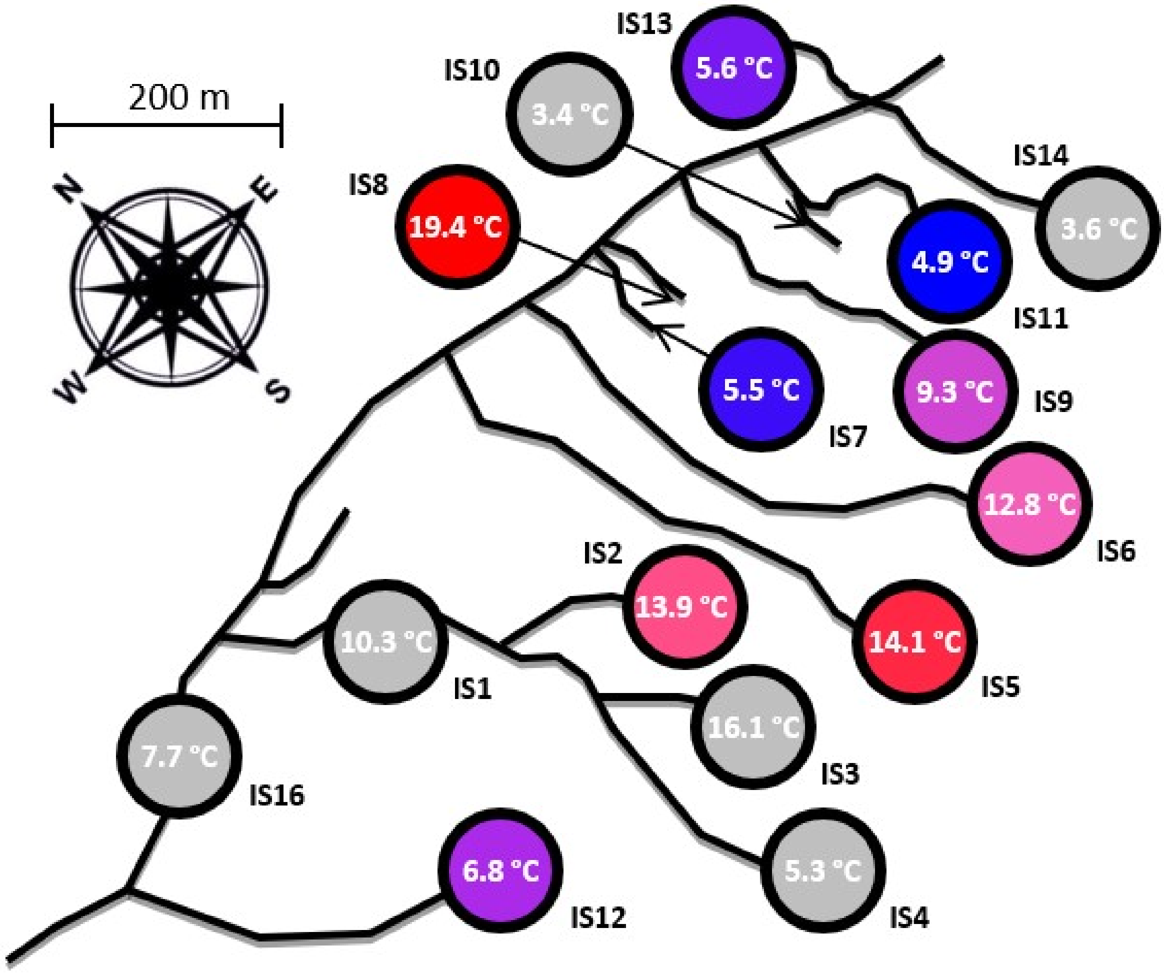

Supplementary Figure 2. Stream temperature profiles for the duration of the study. Temperatures were logged every four hours from 30th April 2015 to 5th July 2017, using Maxim Integrated DS1921G Thermochron iButton temperature loggers. Note that the loggers in IS7 and IS4 were not recovered during summer sampling in 2016 and 2017, respectively, so there are no data for the preceding 12 months. Colours and stream codes are explained in Supplementary Figure 1.

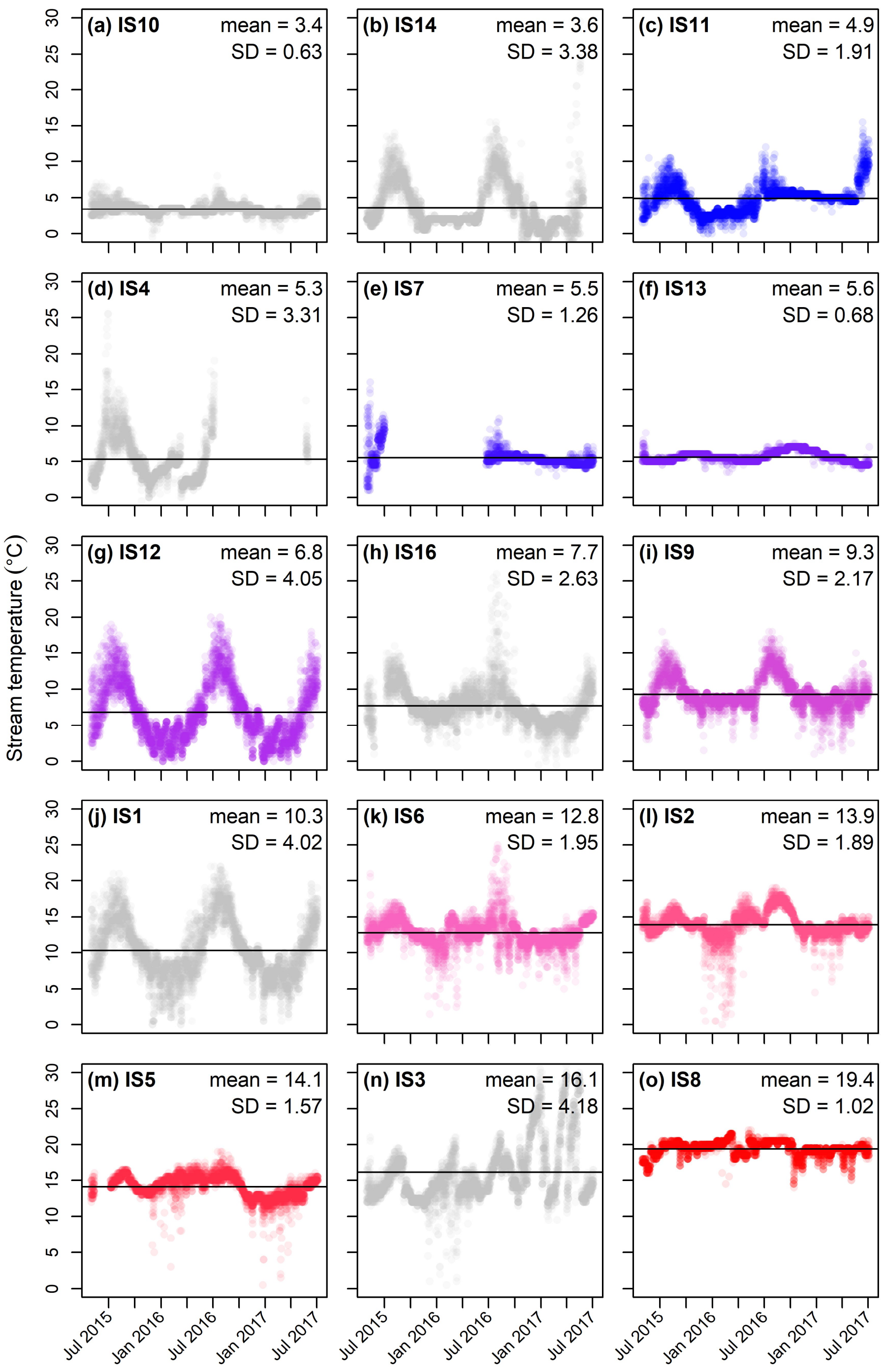

Supplementary Figure 3. Semivariogram exploring potential spatial autocorrelation in the residuals of the best-fitting model. The semivariogram represents the squared difference between all pairwise residual data points as a function of the distance between the two points. The lack of a clear pattern indicates no evidence for spatial autocorrelation, which is further supported by a Moran's *I* test on the model residuals and a Mantel test for spatial autocorrelation in the temperature gradient (see *Exploration of spatial autocorrelation* section in the *Methods*).

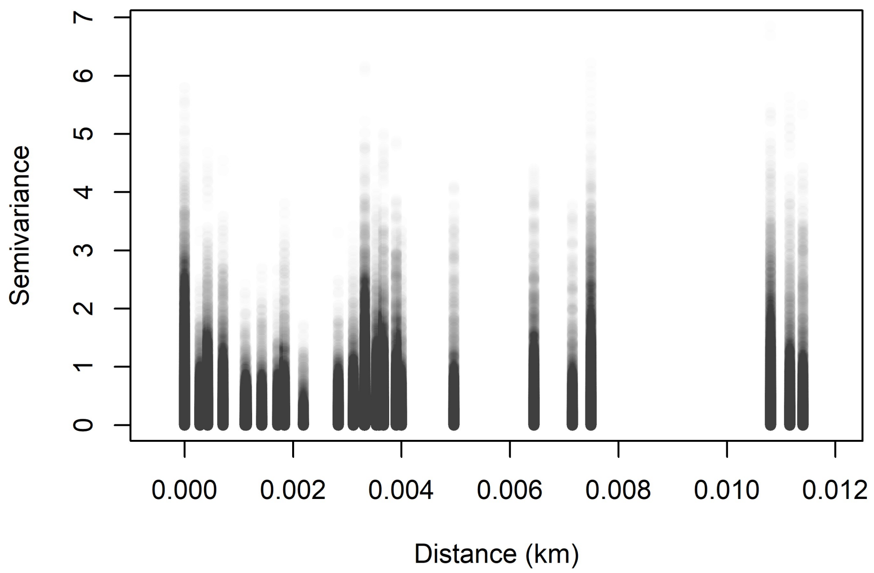

Supplementary Figure 4. Evolution of the natural logarithm of metabolic rate across the time-calibrated phylogeny of the 16 species in the final dataset. Given that there were multiple measurements for any given species, the values shown here in different colours are averages. The values of ancestral nodes were estimated from a phylogenetically-corrected intercept-only model, fitted with MCMCglmm (see *Exploration of phylogenetic structure* section in the *Methods*). Nodes marked with a rhombus are those that we calibrated using age estimates from the TimeTree database. The figure was rendered using the '*ggtree*' R package[8] (v. 3.2.1). The mean posterior phylogenetic heritability estimate of the natural logarithm of metabolic rate was 0.48. This means that nearly half of the variation can be explained by the gradual evolution of metabolic rate across the phylogeny, with the other half arising from other sources including (but not necessarily limited to) acclimation and measurement error.

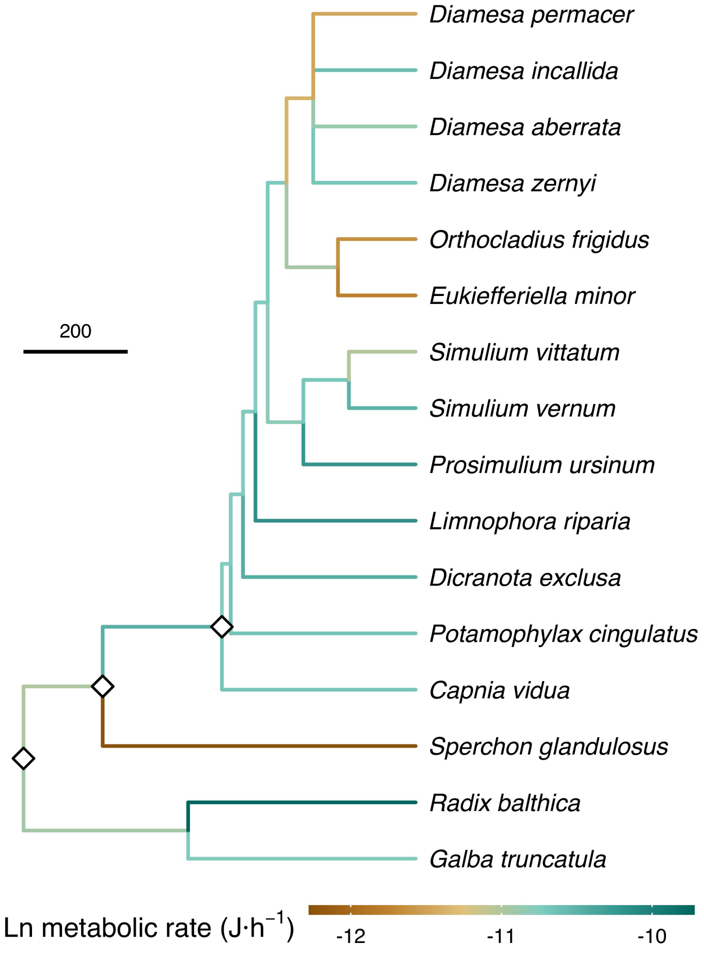

Supplementary Figure 5. Chronic exposure to warmer conditions alters the size- and temperature-dependence of metabolic rate, even after considering phylogenetic information. (**a**) Temperature-corrected metabolic rates are elevated for smaller organisms and suppressed for larger organisms after chronic exposure to warmer conditions, seen as (**b**) a decline in their allometric scaling exponent. The dashed line is the three-quarter scaling expected from MTE. (**c**) Mass-corrected metabolic rates are suppressed at lower acute temperatures and elevated at higher acute temperatures after chronic exposure to warmer conditions, seen as (**d**) an increase in their activation energy (thermal sensitivity). The dashed line is the typical activation energy of 0.65 eV expected from MTE for heterotrophic metabolism. The bars in **b** and **d** represent standard error around the mean from partial residual analysis of linear mixed effects models fitted in **a** and **c**, respectively. Colours of points and lines in all panels indicate the environmental temperature to which species have been chronically exposed (see graphical legends). See *Exploration of phylogenetic structure* section in the *Methods* for more details.

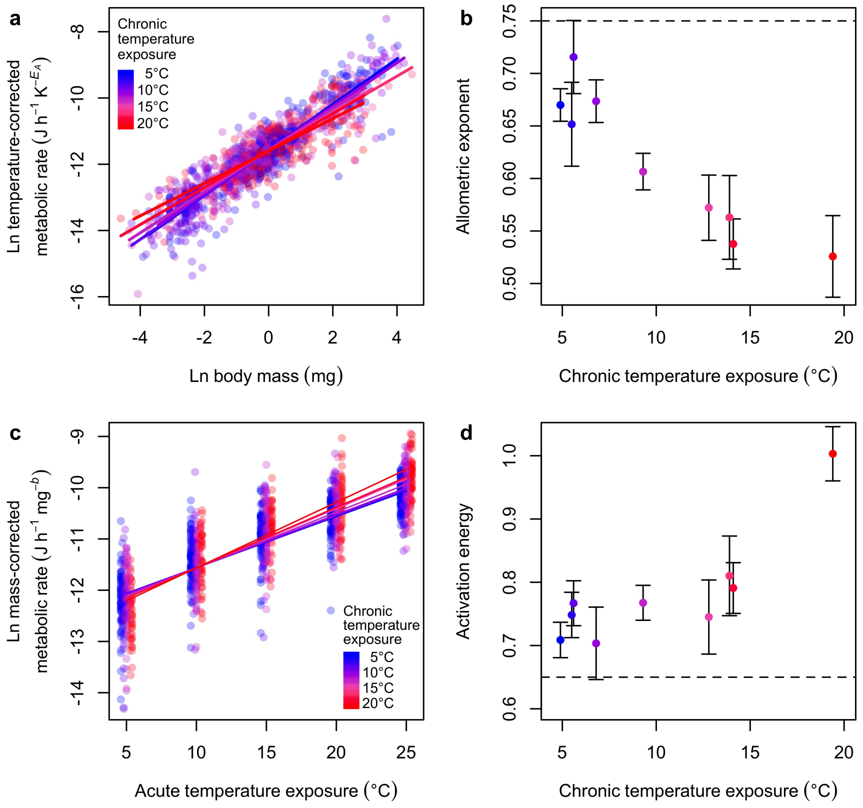

Supplementary Figure 6. Model-estimated total energy flux can qualitatively predict empirical measurements of ecosystem respiration in the study streams. The lines of best fit show the relationships for models with (Linear regression: $F_{1,9} = 25.04$, $y = 1.400 + 0.608\,x$, $r^2 = 0.71$) and without (Linear regression: $F_{1,9} = 24.61$, $y = 1.440 + 0.639\,x$, $r^2 = 0.70$) metabolic plasticity. The shaded areas are the 95% prediction bounds. There is little difference in the explanatory power of the two models here because there is no warming scenario involved (just chronic exposure to different stream temperatures), and thus little scope for metabolic plasticity to alter energy flux. See Supplementary Discussion above for an explanation of the quantitative difference between the two variables.

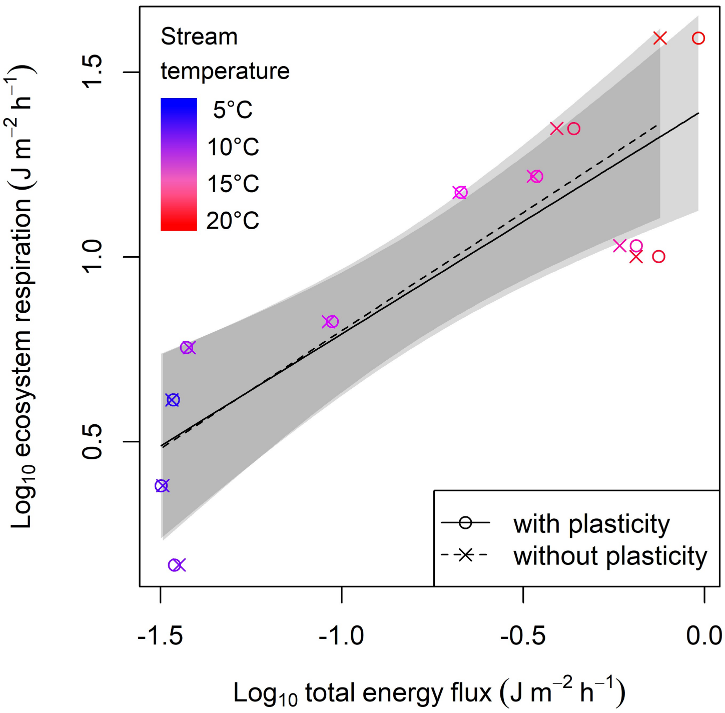

Supplementary Figure 7. Chronic exposure to warmer conditions alters the size- and temperature-dependence of metabolic rate, even after considering the standard deviation of chronic temperature exposure as a random effect. (**a**) Temperature-corrected metabolic rates are elevated for smaller organisms and suppressed for larger organisms after chronic exposure to warmer conditions, seen as (**b**) a decline in their allometric scaling exponent. The dashed line is the three-quarter scaling expected from MTE. (**c**) Mass-corrected metabolic rates are suppressed at lower acute temperatures and elevated at higher acute temperatures after chronic exposure to warmer conditions, seen as (**d**) an increase in their activation energy (thermal sensitivity). The dashed line is the typical activation energy of 0.65 eV expected from MTE for heterotrophic metabolism. The bars in **b** and **d** represent standard error around the mean from partial residual analysis of linear mixed effects models fitted in **a** and **c**, respectively. Colours of points and lines in all panels indicate the environmental temperature to which species have been chronically exposed (see graphical legends).

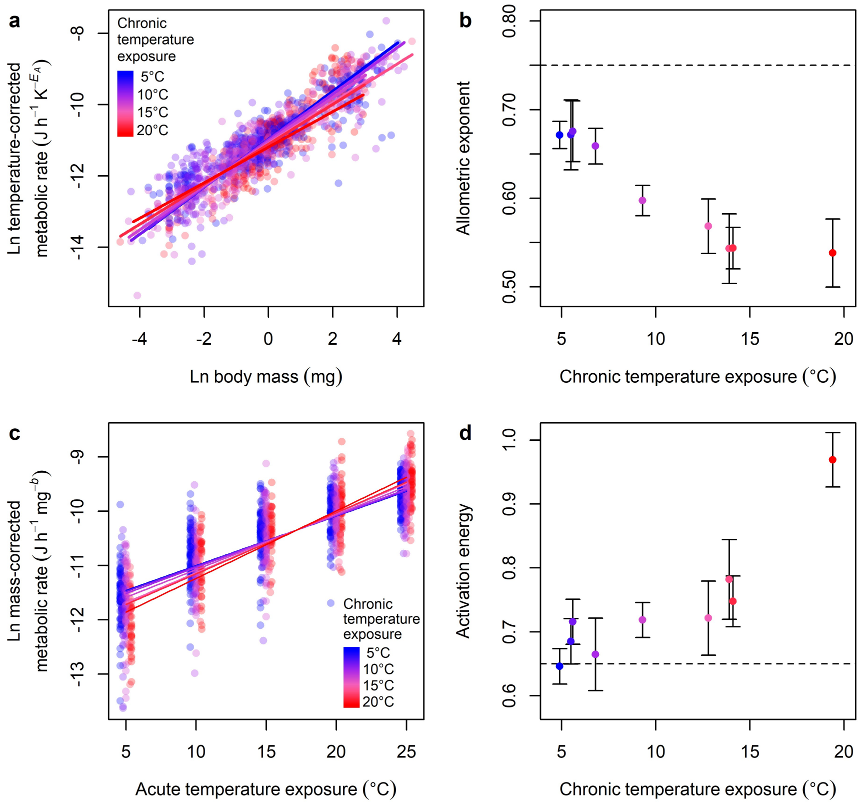

Supplementary Figure 8. Size-dependence of metabolic rate for individual species. The linear regression between log body mass and log metabolic rate is visualised for each species × stream combination (n = 44 populations), after temperature-correcting metabolic rates using the activation energies from the Metabolic Theory of Ecology models specified in Supplementary Table 5. Relationships for the same species from different streams are included in the same plot for visual comparison, but analysed separately. The colour of the data points and regression lines corresponds to the long-term mean temperature of each stream (see Supplementary Figure 2).

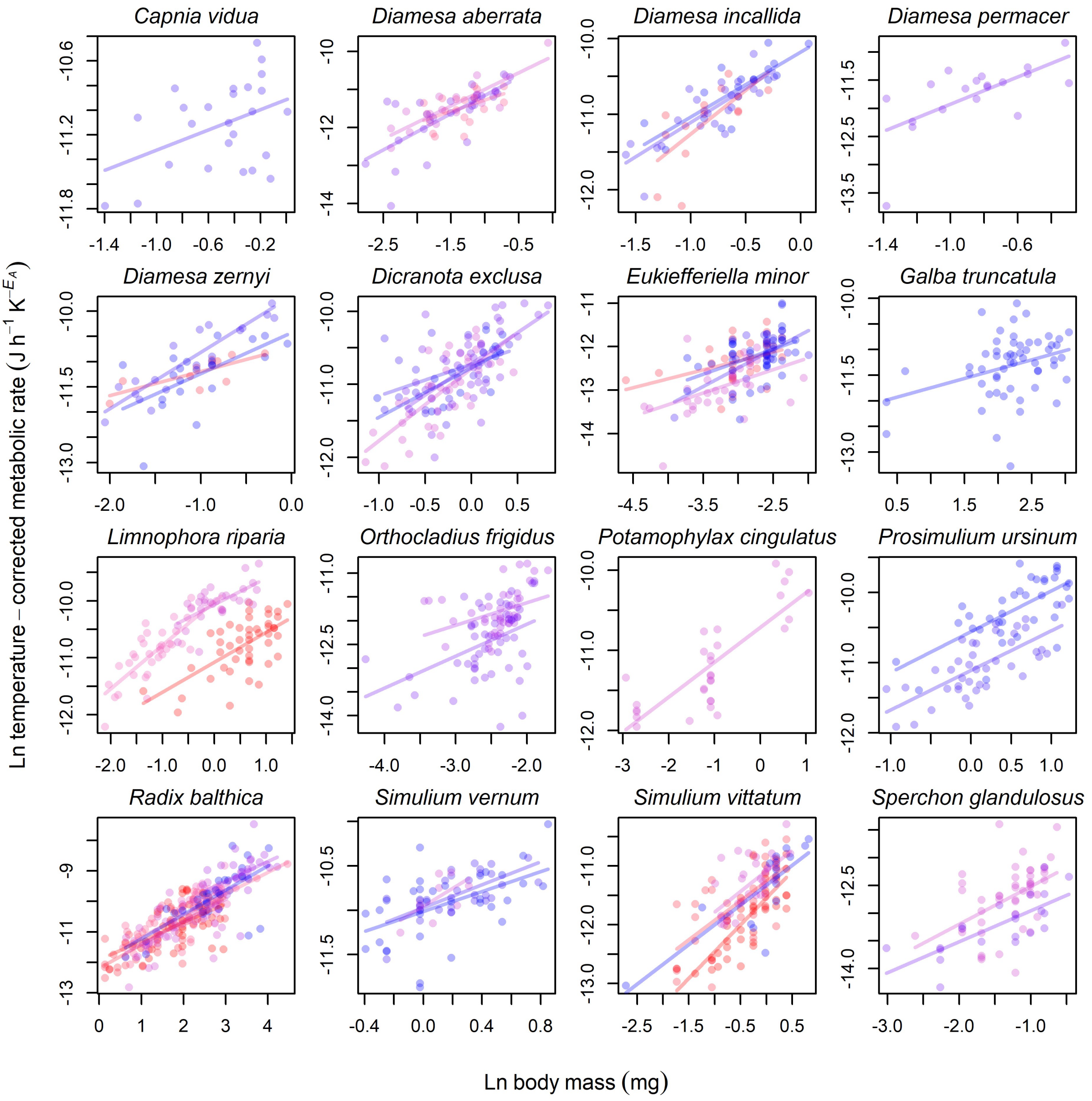

Supplementary Figure 9. Temperature-dependence of metabolic rate for individual species. The linear regression between temperature and log metabolic rate is visualised for each species × stream combination (n = 44 populations), after mass-correcting metabolic rates using the allometric exponents from the Metabolic Theory of Ecology models specified in Supplementary Table 5. Relationships for the same species from different streams are included in the same plot for visual comparison, but analysed separately. The colour of the data points and regression lines corresponds to the long-term mean temperature of each stream (see Supplementary Figure 2).

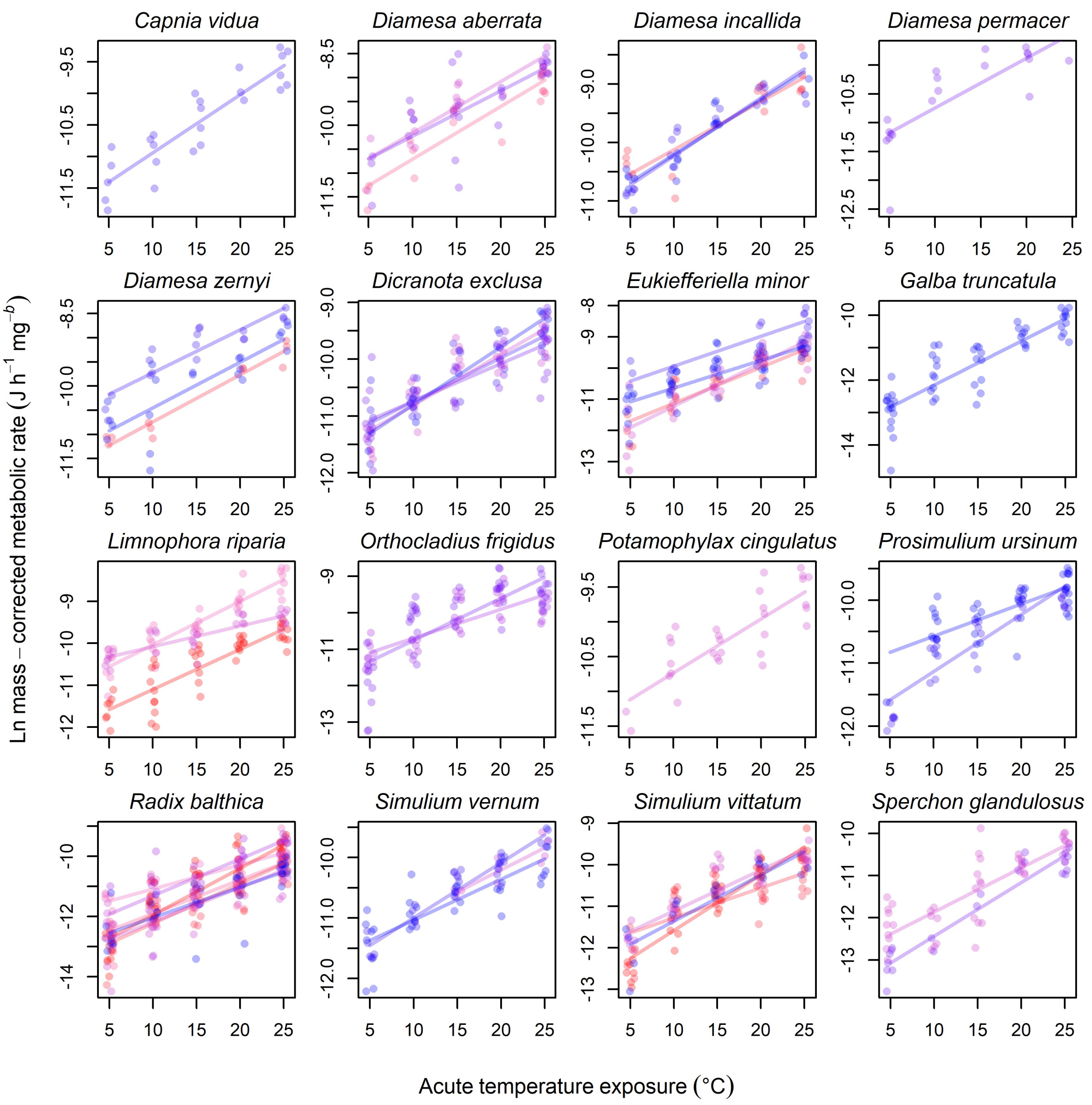

Supplementary Figure 10. Chronic exposure to warmer conditions alters the size- and temperature-dependence of metabolic rate for the full dataset (*n* = 1,819 individuals). (**a**) Temperature-corrected metabolic rates are elevated for smaller organisms and inhibited for larger organisms after chronic exposure to warmer conditions, seen as (**b**) a decline in their allometric scaling exponent. (**c**) Mass-corrected metabolic rates are inhibited at lower acute temperatures and elevated at higher acute temperatures after chronic exposure to warmer conditions, seen as (**d**) an increase in their activation energy. The bars in **b** and **d** represent standard error around the mean from partial residual analysis of linear mixed effects models fitted in **a** and **c**, respectively (see Supplementary Table 6 for model parameters and the Supplementary Note above for underlying R code). The colour of points in all panels relates to the extent of chronic temperature exposure they have experienced (see graphical legends).

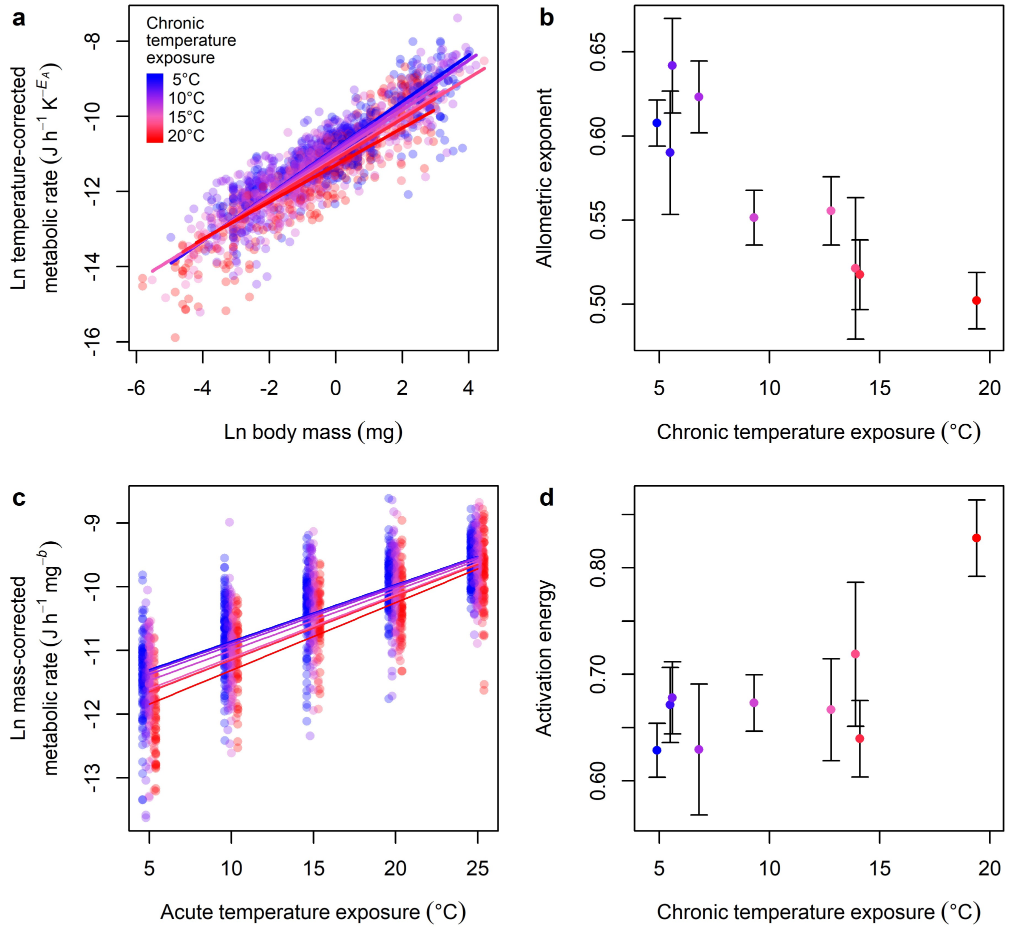

Supplementary Table 1. Length-mass relationships used for estimating body mass of invertebrates. Linear dimensions (HW = head capsule width; BL = body length; SL = shell length; BW = body width), reference to published papers containing length-dry mass relationships, the taxonomic groups measured in the referenced study, and regression equations that correspond to each species (x = linear dimension in mm; y = dry mass in mg) are also provided.

Species	Dimension	Reference	Group	Formula
Capnia vidua	HW	[9]	*Allocapnia* spp.	$y = 0.5438x^{3.255}$
Diamesa aberrata	HW	[10]	Chironomidae	$y = 4.86x^{3.15}$
Diamesa incallida	HW	[10]	Chironomidae	$y = 4.86x^{3.15}$
Diamesa permacer	HW	[10]	Chironomidae	$y = 4.86x^{3.15}$
Diamesa zernyi	HW	[10]	Chironomidae	$y = 4.86x^{3.15}$
Dicranota exclusa	BL	[9]	*Dicranota* sp.	$y = 0.0027x^{2.637}$
Eukiefferiella minor	HW	[11]	Orthocladiinae	$y = 0.8694x^{1.72}$
Galba truncatula	SL	[12]	*Radix balthica*	$y = 0.1002x^{2.6575}$
Limnophora riparia	BL	[13]	*Limnophora riparia*	$y = 0.00056x^{3.4032}$
Orthocladius frigidus	HW	[11]	Orthocladiinae	$y = 0.8694x^{1.72}$
Potamophylax cingulatus	HW	[14]	*Potamophylax* spp.	$y = 1.872x^{3.6358}$
Prosimulium ursinum	BL	[12]	Simuliidae	$y = 0.0025x^{3.0676}$
Radix balthica	SL	[12]	*R. balthica*	$y = 0.1002x^{2.6575}$
Simulium vernum	BL	[12]	Simuliidae	$y = 0.0025x^{3.0676}$
Simulium vittatum	BL	[12]	Simuliidae	$y = 0.0025x^{3.0676}$
Sperchon glandulosus	BW	[15]	*Sperchon glandulosus*	$y = 0.185x^{2.599}$

Supplementary Table 2. Nucleotide sequences corresponding to the 16 species in the final dataset. Nucleotide sequences of the 5' region of the cytochrome c oxidase subunit I gene (COI-5P) were extracted from the Barcode of Life Data System database for use in phylogenetic analysis (see *Exploration of phylogenetic structure* section in the *Methods*). Note that no sequence could be found for *Diamesa permacer*.

Species	BOLD Sequence ID
Capnia vidua	FBAQU1306-12
Diamesa aberrata	ATNA099-09
Diamesa incallida	CHIFI036-16
Diamesa permacer	-
Diamesa zernyi	GBDP45129-19
Dicranota exclusa	FINTI316-12
Eukiefferiella minor	EBACH086-16
Galba truncatula	GBMIN103698-17
Limnophora riparia	GBDP5802-09
Orthocladius frigidus	EBACH118-16
Potamophylax cingulatus	BHMKK238-12
Prosimulium ursinum	FISIM014-11
Radix balthica	GBMIN110649-17
Simulium vernum	ACBB329-07
Simulium vittatum	RRSSC3810-15
Sperchon glandulosus	ACHAR464-18

Supplementary Table 3. Statistical output for the optimal model exploring the key drivers of metabolic rate, incorporating information on phylogenetic structure. The estimated coefficients (value) for size- and temperature-dependence parameters are shown with lower 95% credible intervals (LCI), upper 95% credible intervals (UCI), and *pMCMC* values, obtained from the MCMCglmm model fitted to metabolic rate data on 44 invertebrate populations from nine streams of different temperature (Supplementary Figure 5). Log metabolic rate [ln(I) in J h^{-1}] was the dependent variable and the random effects structure included a random intercept for species identity and random slopes for each of the main effects. The most parsimonious model (ΔDIC > 19) included an intercept [ln(I_0)], main effects of log body mass [ln(M) in mg], acute temperature exposure [T_A in K], and chronic temperature exposure [T_C in K], and interactive effects of T_C on ln(M) and T_A. See *Exploration of phylogenetic structure* section in the *Methods* for more details.

	**Value**	**LCI**	**UCI**	***pMCMC* value**
I_0	-11.55	-12.30	-10.820	<0.001
ln(M)	0.606	0.301	0.893	<0.001
T_S	0.791	0.638	0.944	<0.001
T_L	-0.010	-0.458	0.415	0.916
ln(M):T_L	-0.091	-0.159	-0.021	0.010
T_S:T_L	0.102	0.057	0.152	<0.001

Supplementary Table 4. Statistical output of models exploring the key drivers of metabolic rate after considering the standard deviation of chronic temperature exposure as a random effect. The estimated coefficients (value) for size- and temperature-dependence parameters are shown with standard errors (SE), t values, and p values, obtained from linear mixed effects models fitted to metabolic rate data on 44 invertebrate populations from nine streams of different temperature (Supplementary Figure 7). In both models, log metabolic rate [$\ln(I)$ in J h^{-1}] was the dependent variable and the random effects structure included a random intercept for species identity and random slopes for each of the main effects. The most parsimonious model contained the full random effects structure (an intercept and slopes for log body mass [$\ln(M)$ in mg], acute temperature exposure [T_A in K], chronic temperature exposure [T_C in K], and the standard deviation of T_C), while the fixed effects included an intercept [$\ln(I_0)$], main effects of $\ln(M)$, T_A, and T_C, and interactive effects of T_C on $\ln(M)$ and T_A. There was similar statistical support ($\Delta AIC = 0.6$) for a model containing an additional interaction between $\ln(M)$ and T_A, but that term was not significant ($t = -1.660$; $p = 0.0972$).

	Value	SE	t value	p value
I_0	-11.06	0.1545	-71.56	<0.0001
$\ln(M)$	0.6084	0.0448	13.58	<0.0001
T_S	0.7311	0.0298	24.57	<0.0001
T_L	-0.1551	0.0560	-2.770	0.0057
$\ln(M)$:T_L	-0.0592	0.0192	-3.093	0.0020
T_S:T_L	0.1096	0.0228	4.800	<0.0001

Supplementary Table 5. Statistical output of Metabolic Theory of Ecology (MTE) models for each population (i.e. species × stream combination) in the study system. Species names are provided, along with the stream they were collected from and its average temperature (Temp) over the study period. The model-estimated value, with associated standard error (SE) and p values, are provided for the intercept (I_0), allometric exponent (b), and activation energy (E) of the MTE model (see Equation 1 in the main text). The r^2 value and number (n) of individual organisms quantified for each model is also provided. The final column indicates whether a population was excluded, which happened if $p > 0.05$ for any term in the model or $r^2 < 0.5$. Note that we only included data where $n \geq 10$ for any given population. Only data that were not excluded following these procedures were analysed for impacts of chronic warming in this study. Relationships are visualised in Supplementary Figures 8-9.

			I_0			b			E					
**Species**	**Stream**	**Temp**	**Value**	**SE**	***p* value**	**Value**	**SE**	***p* value**	**Value**	**SE**	***p* value**	r^2	n	**Exclude**
Capnia vidua	7	5.5	-10.91	0.135	<0.001	0.41	0.194	0.046	0.66	0.069	<0.001	0.82	25	no
Diamesa aberrata	2	13.9	-10.68	0.410	<0.001	0.60	0.262	0.035	0.79	0.084	<0.001	0.87	21	no
Diamesa aberrata	9	9.3	-10.13	0.211	<0.001	0.87	0.147	<0.001	0.77	0.109	<0.001	0.78	17	no
Diamesa aberrata	13	5.6	-10.20	0.383	<0.001	0.95	0.204	<0.001	0.68	0.128	<0.001	0.72	27	no
Diamesa incallida	5	14.1	-10.10	0.296	<0.001	1.16	0.319	0.003	0.59	0.084	<0.001	0.81	16	no
Diamesa incallida	7	5.5	-10.20	0.140	<0.001	0.91	0.154	<0.001	0.71	0.063	<0.001	0.87	22	no
Diamesa incallida	11	4.9	-10.18	0.113	<0.001	0.86	0.167	<0.001	0.67	0.066	<0.001	0.87	18	no
Diamesa permacer	13	5.6	-10.72	0.345	<0.001	1.20	0.367	0.005	0.62	0.121	<0.001	0.73	19	no
Diamesa zernyi	5	14.1	-10.72	0.162	<0.001	0.48	0.149	0.012	0.70	0.063	<0.001	0.92	11	no
Diamesa zernyi	7	5.5	-9.70	0.170	<0.001	1.11	0.124	<0.001	0.64	0.079	<0.001	0.90	18	no
Diamesa zernyi	11	4.9	-10.42	0.299	<0.001	0.81	0.256	0.005	0.68	0.111	<0.001	0.71	21	no

Dicranota exclusa	7	5.5	-10.72	0.102	<0.001	0.43	0.198	0.039	0.55	0.076	<0.001	0.73	24	no
Dicranota exclusa	9	9.3	-10.78	0.090	<0.001	0.99	0.153	<0.001	0.64	0.055	<0.001	0.85	38	no
Dicranota exclusa	11	4.9	-10.77	0.103	<0.001	0.68	0.199	0.006	0.72	0.072	<0.001	0.90	14	no
Dicranota exclusa	13	5.6	-10.72	0.078	<0.001	0.85	0.181	<0.001	0.47	0.065	<0.001	0.57	43	no
Eukiefferiella claripennis	8	19.4	-11.94	2.197	0.001	0.35	0.487	0.491	0.93	0.207	0.002	0.72	11	yes
Eukiefferiella minor	5	14.1	-11.10	0.553	<0.001	0.41	0.175	0.026	0.82	0.084	<0.001	0.80	32	no
Eukiefferiella minor	7	5.5	-9.92	0.789	<0.001	0.86	0.282	0.010	0.68	0.111	<0.001	0.79	15	no
Eukiefferiella minor	8	19.4	-12.11	0.876	<0.001	0.25	0.199	0.223	0.75	0.209	0.002	0.46	26	yes
Eukiefferiella minor	9	9.3	-11.18	0.569	<0.001	0.54	0.175	0.004	0.98	0.089	<0.001	0.79	34	no
Eukiefferiella minor	11	4.9	-10.62	0.589	<0.001	0.58	0.204	0.007	0.63	0.079	<0.001	0.72	47	no
Galba truncatula	9	9.3	-11.82	0.425	<0.001	0.11	0.253	0.682	0.94	0.106	<0.001	0.84	19	yes
Galba truncatula	11	4.9	-12.11	0.316	<0.001	0.36	0.141	0.013	0.97	0.077	<0.001	0.76	56	no
Limnophora riparia	5	14.1	-10.23	0.145	<0.001	0.24	0.134	0.084	0.28	0.051	<0.001	0.42	41	yes
Limnophora riparia	6	12.8	-10.01	0.133	<0.001	0.77	0.112	<0.001	0.74	0.057	<0.001	0.84	35	no
Limnophora riparia	7	5.5	-10.17	0.060	<0.001	0.18	0.096	0.086	0.51	0.042	<0.001	0.89	19	yes
Limnophora riparia	8	19.4	-11.08	0.089	<0.001	0.53	0.099	<0.001	0.69	0.059	<0.001	0.80	46	no
Limnophora riparia	9	9.3	-10.06	0.054	<0.001	0.46	0.101	<0.001	0.35	0.044	<0.001	0.79	31	no
Limnophora riparia	11	4.9	-10.78	0.104	<0.001	0.20	0.176	0.264	0.49	0.071	<0.001	0.61	44	yes
Macropelopia sp	13	5.6	-10.31	0.353	<0.001	1.12	0.562	0.078	0.47	0.258	0.101	0.43	12	yes
Orthocladius frigidus	12	6.8	-10.67	0.458	<0.001	0.47	0.188	0.016	0.57	0.069	<0.001	0.62	44	no
Orthocladius frigidus	13	5.6	-10.73	0.535	<0.001	0.67	0.207	0.002	0.82	0.097	<0.001	0.61	51	no
Orthocladius oblidens	8	19.4	-11.29	0.762	<0.001	0.43	0.281	0.158	0.96	0.126	<0.001	0.83	13	yes
Potamophylax cingulatus	9	9.3	-10.71	0.114	<0.001	0.43	0.059	<0.001	0.56	0.075	<0.001	0.79	30	no
Potamophylax cingulatus	11	4.9	-7.65	0.751	<0.001	-0.09	0.245	0.704	0.56	0.122	<0.001	0.34	41	yes
Prosimulium ursinum	7	5.5	-11.11	0.060	<0.001	0.56	0.091	<0.001	0.65	0.046	<0.001	0.87	40	no
Prosimulium ursinum	11	4.9	-10.56	0.116	<0.001	0.58	0.110	<0.001	0.36	0.067	<0.001	0.57	36	no

Radix balthica	2	13.9	-11.88	0.182	<0.001	0.71	0.092	<0.001	0.80	0.089	<0.001	0.83	31	no
Radix balthica	5	14.1	-12.19	0.171	<0.001	0.78	0.100	<0.001	0.94	0.080	<0.001	0.86	41	no
Radix balthica	6	12.8	-11.06	0.548	<0.001	0.48	0.211	0.031	0.57	0.072	<0.001	0.68	31	no
Radix balthica	8	19.4	-11.91	0.392	<0.001	0.61	0.176	0.001	1.11	0.102	<0.001	0.75	40	no
Radix balthica	9	9.3	-12.15	0.212	<0.001	0.74	0.101	<0.001	0.82	0.069	<0.001	0.76	59	no
Radix balthica	11	4.9	-12.01	0.405	<0.001	0.79	0.141	<0.001	0.73	0.101	<0.001	0.71	34	no
Radix balthica	12	6.8	-11.27	1.004	<0.001	0.65	0.293	0.038	0.87	0.135	<0.001	0.64	26	no
Simulium vernum	7	5.5	-10.97	0.067	<0.001	0.65	0.162	0.001	0.65	0.041	<0.001	0.91	27	no
Simulium vernum	9	9.3	-10.96	0.149	<0.001	1.01	0.424	0.044	0.54	0.096	<0.001	0.77	11	no
Simulium vernum	11	4.9	-11.01	0.071	<0.001	0.56	0.174	0.003	0.48	0.060	<0.001	0.72	36	no
Simulium vittatum	5	14.1	-11.26	0.146	<0.001	0.66	0.137	<0.001	0.51	0.098	<0.001	0.60	32	no
Simulium vittatum	6	12.8	-11.60	0.160	<0.001	0.29	0.124	0.032	0.48	0.129	0.002	0.39	22	yes
Simulium vittatum	8	19.4	-11.55	0.080	<0.001	0.91	0.108	<0.001	0.96	0.059	<0.001	0.89	41	no
Simulium vittatum	9	9.3	-11.09	0.079	<0.001	0.70	0.152	<0.001	0.70	0.058	<0.001	0.81	35	no
Simulium vittatum	11	4.9	-11.33	0.135	<0.001	0.67	0.143	<0.001	0.80	0.122	<0.001	0.84	16	no
Sperchon glandulosus	9	9.3	-11.83	0.271	<0.001	0.68	0.190	0.001	0.75	0.079	<0.001	0.71	47	no
Sperchon glandulosus	13	5.6	-12.40	0.223	<0.001	0.56	0.127	<0.001	0.91	0.071	<0.001	0.93	21	no
Thienemanniella sp	13	5.6	-13.42	1.208	<0.001	-0.49	0.329	0.157	-0.03	0.261	0.920	0.04	18	yes

Supplementary Table 6. Statistical output of models exploring the key drivers of metabolic rate for the full dataset (n = 1,819 individuals). The estimated coefficients (value) for size- and temperature-dependence parameters are shown with standard errors (SE), t values, and p values, obtained from linear mixed effects models fitted to metabolic rate data on invertebrate populations from 9 streams of different temperature (Supplementary Figure 10). In both models, log metabolic rate [ln(I) in J h^{-1}] was the dependent variable and the random effects structure included a random intercept for species identity and random slopes for each of the main effects. (a) The most parsimonious model included an intercept [ln(I_0)], main effects of log body mass [ln(M) in mg], acute temperature exposure [T_A in K], and chronic temperature exposure [T_C in K], and interactive effects of T_C on ln(M) and T_A. (b) An alternative model without metabolic plasticity contains only an intercept and main effects for ln(M) and T_A, in line with the general MTE prediction of a universal size-scaling and activation energy (but with ΔAIC > 9, indicating significantly weaker explanatory power than the model with metabolic plasticity).

Model	Parameter	Value	SE	*t* value	*p* value
(a) With plasticity	I_0	-11.01	0.1188	-92.709	<0.001
	ln(M)	0.5700	0.0451	12.646	<0.001
	T_A	0.6827	0.0278	24.573	<0.001
	T_C	-0.1612	0.0581	-2.773	0.006
	ln(M):T_C	-0.0608	0.0221	-2.748	0.006
	T_A:T_C	0.0534	0.0204	2.624	0.009
(b) Without plasticity	I_0	-11.04	0.1200	-92.022	<0.001
	ln(M)	0.5673	0.0467	12.154	<0.001
	T_A	0.6823	0.0286	23.877	<0.001

Supplementary Table 7. Comparison of random effects structures for the full model (Equation 3 in the main text) used for determining effects of body mass, acute temperature exposure, and chronic temperature exposure on metabolic rate. Model identity (ID) and corresponding random effects structures are defined in the Supplementary Note above. The degrees of freedom (df), Akaike Information Criterion (AIC), and difference in Akaike Information Criterion relative to the most parsimonious model (ΔAIC) are also displayed.

Model ID	Random effects	df	AIC	ΔAIC
r1	none	9	2798.9	791.1
r2	~ 1 | species	10	2138.4	130.5
r3	~ 1 + ln(M) | species	12	2082.5	74.6
r4	~ 1 + T_A | species	12	2093.7	85.8
r5	~ 1 + T_C | species	12	2090.6	82.7
r6	~ 1 + ln(M) + T_A | species	15	2039.0	31.2
r7	~ 1 + ln(M) + T_C | species	15	2053.4	45.5
r8	~ 1 + T_A + T_C | species	15	2042.9	35.0
r9	~ 1 + ln(M) + T_A + T_C | species	19	2007.9	0

Supplementary Table 8. Comparison of fixed effects structures for determining effects of body mass, acute temperature exposure, and chronic temperature exposure on metabolic rate. Model identity (ID) and corresponding fixed effects structures are defined in the Supplementary Note above. The degrees of freedom (df), Akaike Information Criterion (AIC), and difference in Akaike Information Criterion relative to the most parsimonious model (ΔAIC) are also displayed. Note that there was similar support for models *m2* and *m5* (ΔAIC < 2), but the additional term in *m2* [ln(M):T_A] was not significant, so we present the results of *m5*.

Model ID	Fixed effects	df	AIC	ΔAIC
m1	ln(M) + T_A + T_C + ln(M):T_A + ln(M):T_C + T_A:T_C + ln(M):T_A:T_C	19	1965.6	2.1
m2	ln(M) + T_A + T_C + ln(M):T_A + ln(M):T_C + T_A:T_C	18	1963.8	0.2
m3	ln(M) + T_A + T_C + ln(M):T_A + ln(M):T_C	17	1984.1	20.6
m4	ln(M) + T_A + T_C + ln(M):T_A + T_A:T_C	17	1971.4	7.9
m5	ln(M) + T_A + T_C + ln(M):T_C + T_A:T_C	17	1963.6	0
m6	ln(M) + T_A + T_C + ln(M):T_A	16	1993.7	30.1
m7	ln(M) + T_A + ln(M):T_A	15	1991.9	28.4
m8	ln(M) + T_A + T_C + ln(M):T_C	16	1983.9	20.3
m9	ln(M) + T_C + ln(M):T_C	15	2039.7	76.2
m10	ln(M) + T_A + T_C + T_A:T_C	16	1969.6	6.0
m11	T_A + T_C + T_A:T_C	15	2009.1	45.6
m12	ln(M) + T_A + T_C	15	1992.6	29.0
m13	ln(M) + T_A	14	1991.2	27.7
m14	ln(M) + T_C	14	2047.8	84.2
m15	T_A + T_C	14	2031.6	68.0
m16	ln(M)	13	2048.0	84.4
m17	T_A	13	2030.1	66.5
m18	T_C	13	2051.4	87.8
m19	none	12	2052.8	89.3

Supplementary References

1 O'Gorman, E. J. *et al.* Temperature effects on fish production across a natural thermal gradient. *Global Change Biology* **22**, 3206-3220 (2016).

2 Gauzens, B. *et al.* fluxweb: An R package to easily estimate energy fluxes in food webs. *Methods in Ecology and Evolution* **10**, 270-279 (2019).

3 O'Gorman, E. J. *et al.* Unexpected changes in community size structure in a natural warming experiment. *Nature Climate Change* **7**, 659-666 (2017).

4 Zuur, A. F., Ieno, E. N., Walker, N. J., Saveliev, A. A. & Smith, G. M. in *Mixed effects models and extensions in ecology with R* 101-142 (Springer, 2009).

5 Bell, T. *et al.* A linear model method for biodiversity–ecosystem functioning experiments. *The American Naturalist* **174**, 836-849 (2009).

6 O'Gorman, E. J. *et al.* A simple model predicts how warming simplifies wild food webs. *Nature Climate Change* **9**, 611-616 (2019).

7 Woodward, G. *et al.* Sentinel systems on the razor's edge: effects of warming on Arctic geothermal stream ecosystems. *Global Change Biology* **16**, 1979-1991 (2010).

8 Yu, G., Smith, D. K., Zhu, H., Guan, Y. & Lam, T. T. Y. ggtree: an R package for visualization and annotation of phylogenetic trees with their covariates and other associated data. *Methods in Ecology and Evolution* **8**, 28-36 (2017).

9 Benke, A. C., Huryn, A. D., Smock, L. A. & Wallace, J. B. Length-Mass Relationships for Freshwater Macroinvertebrates in North America with Particular Reference to the Southeastern United States. *Journal of the North American Benthological Society* **18**, 308-343 (1999).

10 Johnston, T. A. & Cunjak, R. A. Dry mass–length relationships for benthic insects: a review with new data from Catamaran Brook, New Brunswick, Canada. *Freshwater Biology* **41**, 653-674 (1999).

11 Baumgärtner, D. & Rothhaupt, K.-O. Predictive Length–Dry Mass Regressions for Freshwater Invertebrates in a Pre-Alpine Lake Littoral. *International Review of Hydrobiology* **88**, 453-463 (2003).

12 Hannesdóttir, E. R., Gíslason, G. M., Ólafsson, J. S., Ólafsson, Ó. P. & O'Gorman, E. J. Increased stream productivity with warming supports higher trophic levels. *Advances in Ecological Research* **48**, 283-340 (2013).

13 Archer, L. C. *et al.* Consistent temperature dependence of functional response parameters and their use in predicting population abundance. *Journal of Animal Ecology* **88**, 1670-1683 (2019).

14 Meyer, E. The relationship between body length parameters and dry mass in running water invertebrates. *Archiv für Hydrobiologie* **117**, 191-203 (1989).

15 Lindegaard, C. Zoobenthos ecology of Thingvallavatn: vertical distribution, abundance, population dynamics and production. *Oikos*, 257-304 (1992).
