# Supplementary material for: Metabolic plasticity can amplify ecosystem responses to global warming
Source: Nat Commun. 2022 Apr 20;13:2161. doi: 10.1038/s41467-022-29808-1 (PMC9021271; doi:10.1038/s41467-022-29808-1)
Supplement: Supplementary file 3 — Reporting Summary [file 41467_2022_29808_MOESM3_ESM.pdf]

nature portfolio

Corresponding author(s): Eoin J. O'Gorman

Last updated by author(s): Mar 5, 2022

Reporting Summary

Nature Portfolio wishes to improve the reproducibility of the work that we publish. This form provides structure for consistency and transparency in reporting. For further information on Nature Portfolio policies, see our Editorial Policies and the Editorial Policy Checklist.

Statistics

For all statistical analyses, confirm that the following items are present in the figure legend, table legend, main text, or Methods section.

n/a	Confirmed	
☐	☒	The exact sample size (*n*) for each experimental group/condition, given as a discrete number and unit of measurement
☐	☒	A statement on whether measurements were taken from distinct samples or whether the same sample was measured repeatedly
☐	☒	The statistical test(s) used AND whether they are one- or two-sided<br>*Only common tests should be described solely by name; describe more complex techniques in the Methods section.*
☐	☒	A description of all covariates tested
☐	☒	A description of any assumptions or corrections, such as tests of normality and adjustment for multiple comparisons
☐	☒	A full description of the statistical parameters including central tendency (e.g. means) or other basic estimates (e.g. regression coefficient) AND variation (e.g. standard deviation) or associated estimates of uncertainty (e.g. confidence intervals)
☐	☒	For null hypothesis testing, the test statistic (e.g. *F*, *t*, *r*) with confidence intervals, effect sizes, degrees of freedom and *P* value noted<br>*Give P values as exact values whenever suitable.*
☒	☐	For Bayesian analysis, information on the choice of priors and Markov chain Monte Carlo settings
☒	☐	For hierarchical and complex designs, identification of the appropriate level for tests and full reporting of outcomes
☒	☐	Estimates of effect sizes (e.g. Cohen's *d*, Pearson's *r*), indicating how they were calculated

Our web collection on statistics for biologists contains articles on many of the points above.

Software and code

Policy information about availability of computer code

Data collection	Dissolved oxygen concentrations and associated oxygen consumption over the duration of each experiment were recorded using the SensorTrace Rate software accompanying the Unisense MicroRespiration equipment used to measure metabolic rates. These rates were transcribed to a Microsoft Excel spreadsheet, along with information on the date, time, experimental chamber, experimental temperature, and a unique organism code which was also written on the sample tube it was preserved in for later identification and body mass estimation.
Data analysis	All statistical analyses were carried out in R 4.0.2, with the following packages: stats, nlme, vegan, fossil, rotl, MCMCglmm, and fluxweb. Statistical R code is provided in the Supplementary Note and full R code generated in this study has been deposited with the University of Essex Research Data Repository under the following DOI: 10.5526/ERDR-00000148

For manuscripts utilizing custom algorithms or software that are central to the research but not yet described in published literature, software must be made available to editors and reviewers. We strongly encourage code deposition in a community repository (e.g. GitHub). See the Nature Portfolio guidelines for submitting code & software for further information.

Data

Policy information about availability of data

All manuscripts must include a data availability statement. This statement should provide the following information, where applicable:

- Accession codes, unique identifiers, or web links for publicly available datasets
- A description of any restrictions on data availability
- For clinical datasets or third party data, please ensure that the statement adheres to our policy

The metabolic rate and energy flux data generated in this study have been deposited with the University of Essex Research Data Repository under the following DOI: 10.5526/ERDR-00000148

Field-specific reporting

Please select the one below that is the best fit for your research. If you are not sure, read the appropriate sections before making your selection.

☐ Life sciences ☐ Behavioural & social sciences ☒ Ecological, evolutionary & environmental sciences

For a reference copy of the document with all sections, see nature.com/documents/nr-reporting-summary-flat.pdf

Ecological, evolutionary & environmental sciences study design

All studies must disclose on these points even when the disclosure is negative.

Study description	Invertebrates were collected from nine streams spanning a temperature gradient of 5-20 °C in the Hengill geothermal valley, Iceland. Laboratory experiments were carried out to determine the effects of body mass, acute temperature exposure, and chronic temperature exposure on oxygen consumption rates as a measure of metabolic rate. In total, oxygen consumption rates were measured for 1,819 individuals, none of which were ever reused in another experiment, thus every data point in the analysis corresponds to a single new individual.
Research sample	The research sample involved freshwater macroinvertebrates collected from geothermal streams in the Hengill valley, Iceland. These organisms were chosen because they contain multiple populations in a single valley that have been chronically exposed to a broad range of temperatures, enabling us to disentangle the acute and chronic effects of temperature on their metabolic rate in laboratory experiments. The macroinvertebrates included snails (Radix balthica, Galba truncatula), mites (Sperchon glandulosus), and many species of fly larvae (Capnia vidua, Diamesa aberrata, Diamesa incallida, Diamesa permacer, Diamesa zernyi, Dicranota exclusa, Eukiefferiella minor, Limnophora riparia, Orthocladius frigidus, Potamophylax cingulatus, Prosimulium ursinum, Simulium vernum, Simulium vittatum). Collections were conducted throughout the summer over a 4-year period, ensuring the maximum coverage of species and associated body size ranges from the natural system. Collections were solely conducted on the basis of availability in the streams, ensuring the taxonomic composition and size distribution of the experiments was representative of the natural system.
Sampling strategy	No statistical methods were used to predetermine sample size, which was solely based on the availability of macroinvertebrates in the Hengill geothermal streams, ensuring the taxonomic composition and size distribution of the experiments was representative of the natural system. Quality control procedures were conducted on the data to exclude any populations with fewer than 10 individuals or a poor fit of the models to the data, ensuring a sufficient sample size for regression analysis (see Data Exclusions section below for more details).
Data collection	Experiments and calculation of metabolic rates from raw dissolved oxygen data were all conducted by the same person (Rebecca L Kordas) to avoid any issues with experimenter bias. Data from experiments were directly entered into Microsoft Excel spreadsheets.
Timing and spatial scale	Laboratory experiments were all conducted from May to July of 2015-2018, i.e. during summer field expeditions to the Hengill valley in Iceland, with sampling of streams occurring within a 2 km^2 area. Access to the field site is complicated by heavy snow on the ground outside the summer window and constraining experiments to the same time of year ensured that invertebrate development (and thus body size) was similar across years.
Data exclusions	We performed multiple linear regressions on log respiration rate (continuous response variable) as a function of log body mass, acute temperature exposure, and chronic temperature exposure (all continuous explanatory variables) for every population (i.e., species × stream combination) in our dataset. Following these analyses, we excluded populations where $n < 10$ individuals, $r2 < 0.5$, and $p > 0.05$ for any term in the model. This excluded any poor quality species-level data and resulted in 1,359 individuals from 44 populations for further analysis. Note that every metabolic rate was measured for a new individual, ensuring complete independence of measurements across treatments. Thus, our data exclusion procedure ensures at least 10 independent replicate measurements of metabolic rate for every population at every acute temperature exposure.
Reproducibility	We repeated the methodology in July 2021 on samples collected from rivers across a temperature gradient in the Cantabrian Mountains, Spain. We are in the process of analysing these data for a follow-up paper.
Randomization	Field-collected macroinvertebrates were only maintained in aquaria in the laboratory for 2-4 days to ensure their health did not deteriorate during the experiments. Thus, the range of taxa, stream origin, and body sizes conducted on any given day was representative of the current laboratory stock. Organisms were collected from across the full stream temperature gradient each day to ensure taxa used in the experiments were not biased towards any particular chronic temperature exposure. The full range of acute temperature exposures were also conducted in the laboratory each day to avoid any bias towards experimental temperatures. Individuals for each species were randomly allocated to acute temperature exposures to avoid any within-species bias towards experimental temperatures.
Blinding	All organisms were assigned a unique code during experimentation. Species names and associated body masses were only reassigned to the code following estimation of metabolic rates from the raw dissolved oxygen data. This effectively blinded the investigator to the experimental organisms that were being tested to help avoid any unconscious bias towards potential outcomes.

Did the study involve field work? ☒ Yes ☐ No

Field work, collection and transport

Field conditions	Field collections of freshwater invertebrates were conducted from May to July of 2015-2018 in the mountainous Hengill region of southwest Iceland. Air temperatures ranged between 0-20 °C, with frequent rainfall and occasional snow.
Location	Fieldwork was performed in the Hengill geothermal valley, Iceland (N 64°03; W 21°18) at 350-420 metres above sea level.
Access & import/export	Access to the field site was obtained through collaboration with local researchers from the University of Iceland (Prof Gísli Már Gíslason) and the Marine and Freshwater Research Institute (Dr Jón S Ólafsson). Experiments were conducted and invertebrates identified in laboratories at the University of Iceland, thus no permits were needed to export samples.
Disturbance	The study caused minimal disturbance to the field site. Researchers were careful to stick to established walking trails and to avoid excessive trampling of the vegetation or streams during sampling. The sampling protocols were non-invasive, involving hand-collections of most invertebrates from rocks and bryophytes, with surber sampling also used to collect bulk samples for sorting in white trays. Great care was taken not to cross-contaminate streams in the system.

Reporting for specific materials, systems and methods

We require information from authors about some types of materials, experimental systems and methods used in many studies. Here, indicate whether each material, system or method listed is relevant to your study. If you are not sure if a list item applies to your research, read the appropriate section before selecting a response.

Materials & experimental systems

n/a	Involved in the study	
☒	☐	Antibodies
☒	☐	Eukaryotic cell lines
☒	☐	Palaeontology and archaeology
☐	☒	Animals and other organisms
☒	☐	Human research participants
☒	☐	Clinical data
☒	☐	Dual use research of concern

Methods

n/a	Involved in the study	
☒	☐	ChIP-seq
☒	☐	Flow cytometry
☒	☐	MRI-based neuroimaging

Animals and other organisms

Policy information about studies involving animals; ARRIVE guidelines recommended for reporting animal research

Laboratory animals	No laboratory animals were used in the study
Wild animals	No wild animals were used in the study
Field-collected samples	Invertebrates were collected from nine streams spanning a temperature gradient of 5-20 °C in the Hengill geothermal valley, Iceland. Organisms were stored in containers within their 'home stream' until the end of each collection day, when they were transported within one hour to the University of Iceland and then transferred into 2 L aquaria filled with water from the main river in Hengill, the Hengladalsá. The aquaria were continuously aerated in temperature-controlled chambers set to the home-stream temperature of the organisms and a 12:12h light:dark cycle. The organisms were maintained without food for at least 24 hours to standardise their digestive state prior to metabolic measurements. Following each experiment, individuals were preserved in 70% ethanol and later identified to species level under a dissecting or compound microscope.
Ethics oversight	No ethical approval was required because the fieldwork and laboratory experiments exclusively involved freshwater macroinvertebrates, consisting of snails, mites, and many different species of fly larvae.

Note that full information on the approval of the study protocol must also be provided in the manuscript.
